# Gentianaceae Family—Derived Bioactive Compounds—Therapeutic Values and Supporting Role in Inflammation and Detoxification

**DOI:** 10.3390/nu17162619

**Published:** 2025-08-13

**Authors:** Wiktoria Andryszkiewicz, Milena Chmielewska, Julia Ciecierska, Paulina Lenkiewicz, Wiktoria Marciniak, Wiktoria Raczycka, Agata Wojno, Julita Kulbacka, Przemysław Niewiński, Katarzyna Bieżuńska-Kusiak

**Affiliations:** 1 Faculty of Medicine, Wroclaw Medical University, Mikulicza-Radeckiego 5, 50-345 Wroclaw, Poland; wiktoria.andryszkiewicz@student.umw.edu.pl (W.A.); milena.chmielewska@student.umw.edu.pl (M.C.); julia.ciecierska@student.umw.edu.pl (J.C.); paulina.lenkiewicz@student.umw.edu.pl (P.L.); wiktoria.marciniak@student.umw.edu.pl (W.M.); wiktoria.raczycka@student.umw.edu.pl (W.R.); agata.wojno@student.umw.edu.pl (A.W.); 2 Student Research Group No. K148, Faculty of Pharmacy, Wroclaw Medical University, Borowska 211A, 50-556 Wroclaw, Poland; 3 Department of Molecular and Cellular Biology, Faculty of Pharmacy, Wroclaw Medical University, Borowska 211A, 50-556 Wroclaw, Poland; 4 Department of Immunology and Bioelectrochemistry, State Research Institute Centre for Innovative Medicine, LT-08406 Vilnius, Lithuania; 5 Department of Clinical Pharmacology, Faculty of Pharmacy, Wroclaw Medical University, Borowska 211A, 50-556 Wroclaw, Poland; przemyslaw.niewinski@umw.edu.pl

**Keywords:** Gentianaceae, *Gentiana*, gentiopicroside, iridoids, flavonoids, xanthones, hepatoprotection, natural therapy

## Abstract

Herbs from the Gentianaceae family are widely known for their medicinal and pharmacological properties. They were used centuries ago as a part of traditional medicine in China and Tibet. This review aims to draw attention to the potential uses of gentian herbs in treating various diseases, including skin conditions, gastrointestinal and liver disorders, wound healing, rheumatoid arthritis, and diabetes. The aim of our study was to systematically summarize current knowledge about key bioactive compounds present in both roots and aerial parts—such as xanthones, iridoids, and flavonoids—and highlight their pharmacological significance. We also focused on the Gentianaceae family’s usage in complementary and alternative medicine, as well as their anti-inflammatory, anti-melanogenic, anti-ischemic, anti-fibrotic, and antioxidant properties, which can be utilized in the treatment and prevention of dermatological diseases, such as skin cancers. Here, we involve ethnomedicinal knowledge with modern pharmacological data; we also highlight the scientific relevance of gentian-derived compounds in drug development. This review concludes that these species represent a promising source of natural agents, while also underlining the need for further research and conservation strategies to preserve threatened species.

## 1. Introduction

**Figure 1:**
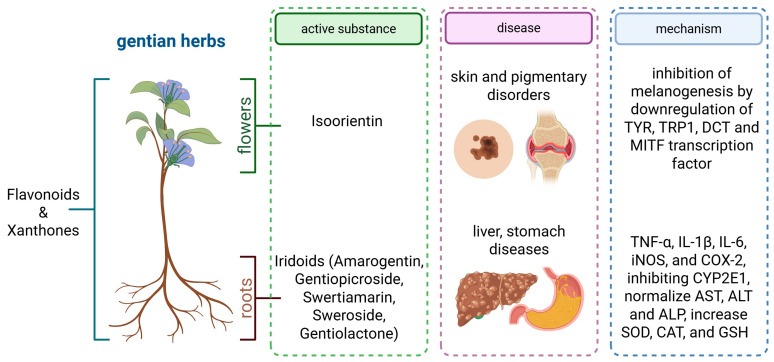
Schematic overview of the principal bioactive constituents of gentian (*Gentiana* spp.) and their proposed therapeutic targets. Specific metabolites are linked to two major indication clusters: (i) skin and pigmentary disorders and (ii) liver and gastric diseases. Mechanistic panels summarize experimentally supported actions: suppression of melanogenesis via downregulation of TYR, TRP-1, DCT, and MITF transcription; and broad anti-inflammatory/detoxifying effects, including reduced TNF-α, IL-1β, IL-6, iNOS, and COX-2 expression, inhibition of CYP2E1, normalization of hepatic enzymes (AST, ALT, ALP), and augmentation of antioxidant defenses (SOD, CAT, GSH). (Created in BioRender. Kulbacka, J. (2025) https://BioRender.com/ys0htas).

Although *Gentiana* accounts for >400 species within the Gentianaceae family, other medicinal genera (e.g., Swertia, Gentianella) share closely related seco-iridoid, xanthone, and flavonoid profiles and are, therefore, covered in this review. Gentians are renowned for their large, trumpet-shaped flowers, which are typically blue [1]. Several species of *Gentiana* are widely used in traditional medicine across various cultures, including those of India, China, and Iran. Shared applications between conventional and modern medicine include anti-inflammatory, hepatoprotective, and diuretic effects. Phytochemical studies reveal that the therapeutic properties of *Gentiana* are attributed to secondary metabolites, including iridoids, flavonoids, and xanthones [2]. Bioactive compounds from the *Gentiana* genus exhibit diverse pharmacological properties through their effects on apoptosis, autophagy, inflammation, nuclear factor kappa-light-chain-enhancer of activated B cells (NF-κB) signaling, mitogen-activated protein kinase (MAPK) pathways, growth factors, and cytochrome P450 enzymes [3]. The medicinal uses of substances extracted from gentian herbs, along with the pharmacological potential of key bioactive compounds, show promise in treating various conditions, including liver disorders, inflammatory disorders, cardiovascular disorders, osteoporosis, thromboembolic disorders, atherosclerosis, and oxidative stress-related damage. Figure 1 below illustrates how gentian-derived metabolites contribute to both anti-inflammatory signaling and metabolic detoxification, supporting their therapeutic value.

## 2. Methodology for Search Strategy and Inclusion Criteria

A structured literature search was conducted from January 2000 to March 2024 using the following databases: PubMed, Web of Science, Scopus, and ScienceDirect. We used Boolean search terms such as: (“Gentiana” OR “Gentianaceae” OR “Swertia”) AND (“phytochemical” OR “bioactive compound” OR “ethnopharmacology”) AND (“anti-inflammatory” OR “hepatoprotective” OR “antioxidant” OR “metabolic disorder” OR “diabetes” OR “traditional medicine”).

Articles were selected if they (1) discussed the chemical composition, pharmacological effects, or ethnomedicinal uses of Gentiana species; (2) were original research, reviews, or authoritative ethnobotanical reports; and (3) provided mechanistic or quantitative data on specific compounds. While we focus on the last two decades, several older references (1991 and 1996) were retained due to their historical or foundational relevance to the traditional use of these plants.

## 3. Development of Gentiana-Based Supplements and Herbal Remedies

Ethnopharmacology has laid the foundation for the therapeutic use of natural compounds found in plants, which have been used and studied for centuries as sources of medicine. Herbs play a significant role in cultural heritage and increasing awareness of the connections between food and health [4].

The scientific name Gentiana originates from King Gentius of Illyria in the second century BCE, who, according to tradition, was the first to recognize the medicinal properties of this plant and used its root in 167 BCE during an epidemic outbreak. In historical medical texts, this name refers to the entire genus and all its species. 

In Iranian traditional medicine, the *Gentiana* species are attributed with numerous medicinal properties, particularly related to their roots. In the past, they were used to treat urinary retention, menstrual disorders, and inflammation of the liver, spleen, and stomach. Additionally, they were used as an antidote for animal venoms. Preparations such as water extracts, vinegar poultices, and honey mixtures were used to treat venomous animal bites, vitiligo, wounds, and sprains. They were also used to induce abortion and treat conjunctivitis [2]. The Gentianaceae family, due to its properties, has found applications in various branches of medicine, and its pharmacological benefits have been widely recognized. One example is gentian violet, also known as crystal violet, a triphenylmethane dye with antibacterial, antifungal, antiparasitic, and antitumor properties. It was first synthesized by Charles Lauth in 1861. Initially used by biologists, it later became popular as an antiseptic under the name pyocyanin in 1891. It was subsequently used in the treatment of sepsis, meningitis, and other diseases.

In the 20th century, gentian violet was widely used to treat infections, burns, thrush, and fungal infections. In a 2010 study, a severe case of eczema caused by group A Streptococcus was successfully treated with oral doxycycline and a 1% gentian violet solution. Gentian violet has also been used for many years to prevent bacterial colonization of the umbilical stump after childbirth. It is an affordable and stable drug with a long history of topical and systemic use, particularly in preventing Chagas disease in South America and as a dermatological agent in developing countries. Its clinical effectiveness arises from two mechanisms of action, which involve inhibiting the nicotinamide adenine dinucleotide phosphate hydrogen (NADPH) oxidase complex in mammalian cells and binding to thioredoxin 2 in infectious organisms. These properties allow gentian violet to support anti-angiogenesis therapies, enhance antitumor immunity, and act as an effective antimicrobial agent in developed countries as well, making it an invaluable treatment [5].

*Gentian* species are widely used in traditional medicine in Europe and the Himalayan region, with applications varying depending on the location. *Gentiana lutea* and *Gentiana cruciata* are particularly valued for their ability to treat loss of appetite, gastrointestinal disorders, liver diseases, and promote wound healing. Due to their valuable properties, they have also been recognized in the European Pharmacopoeia and European Medicines Agency (EMA) guidelines. In some regions, these plants are substituted by other species, such as *Gentiana asclepiadea* and *Gentiana pneumonanthe*, highlighting the versatility of these plants and their importance in traditional medicine [6].

## 4. Overview of Gentian Herbs

### 4.1. Phytochemistry of Gentian Herbs

Studies have shown that in plants of the genus *Gentiana*, we can find numerous bioactive compounds such as xanthones, iridoids, and flavonoids [3].

Iridoids are a class of cyclopentane pyran monoterpenes. They consist of two basic carbon frameworks: substituted iridoids and secoiridoids. Based on their structure, iridoids are categorized into four groups: iridoid glycosides, secoiridoid glycosides, non-glycosidic iridoids, and bis-iridoids. These compounds typically have hemiacetal hydroxyl groups and are biologically active. Most iridoids are glycosides, meaning they are bound to glucose at the C-1 hydroxyl group [7]. In gentiana plants, we can find the following iridoids: gentiopicroside, swertiamarin, sweroside, and amarogentin [3].

Flavonoids are a large family of hydroxylated polyphenolic compounds found in plants. In the human diet, flavonoids are naturally present in fruits, vegetables, and tea [8]. From plants of the genus *Gentiana*, we can isolate flavonoids such as isoorientin and isovitexin [3]. Flavonoids are a diverse group of compounds that are classified into various subgroups based on the attachment of the B ring to the C ring and the degree of unsaturation and oxidation of the C ring [9].

Xanthones are secondary metabolites found in certain plant families, including Gentianaceae, in which we can find substances such as isogentisin, bellidifolin, and mangiferin [3]. They share structural similarities with flavonoids; however, xanthones are less widespread in nature compared to flavonoids. Xanthones are typically present as mono- or polymethyl ethers or glycosides, though they can sometimes occur as polyhydroxylated compounds [8,10]. Table 1 below represents the chemical structures of bioactive molecules from the Gentiana family.

As summarized in Table 1, three phytochemical classes dominate the pharmacological landscape of Gentianaceae: seco-iridoids, flavonoids, and xanthones. Among the seco-iridoids, gentiopicroside, amarogentin, and swertiamarin are both the most abundant (collectively 1–6% of G. lutea root dry weight) and the most frequently investigated, underpinning the family’s well-documented hepatoprotective and anti-inflammatory actions [19,20]. The aerial parts concentrate flavonoids, with isoorientin and isovitexin occurring at 0.2–1% and featuring prominently in studies on melanogenesis inhibition and vascular protection [21]. Although present at lower levels in the roots (0.05–0.3%), the xanthones bellidifolin and isogentisin are the focus of a growing body of neuroprotective and antioxidant research [22,23]. Due to highlighting, these high-abundance or high-visibility molecules focus researchers directly toward the compounds—and bioactivities—most relevant for further pharmacological exploitation.

#### 4.1.1. Amarogentin—Anti-Fibrotic and Antithrombotic Activity

Amarogentin (Table 1) is a secoiridoid glycoside that can be extracted from *Gentiana* roots [24]. The substance promotes apoptosis by increasing the expression of B-cell lymphoma-2 (Bcl-2) and decreasing levels of Bcl-2-associated X protein (Bax). In the course of liver injury, hepatic stellate cells (HSCs) are activated, which produce α-smooth muscle actin (α-SMA) and extracellular matrix (ECM), leading to fibrosis and further liver damage. The proven acceleration of apoptosis of mouse HSCs offers potential as a therapeutic approach for liver fibrosis. In this experiment, amarogentin also caused downregulation of the MAPK signalling pathways, which play an important role in liver fibrosis. Suppression of the MAPK pathway was caused by a dose-dependent decrease in phosphorylation of c-Jun NH2-terminal kinase (JNK), extracellular signal-regulated kinase (ERK), and p38. Amarogentin also inhibits the expression of transforming growth factor-1 (TGF-1) in the mouse model of liver fibrosis discussed above [25]. TGF-1, as a profibrogenic cytokine, is involved in liver fibrosis by influencing the metabolism of extracellular matrix proteins [26]. Amarogentin treatment inhibits in vivo thrombus formation in mice. Furthermore, it inhibits collagen-induced platelet aggregation and also suppresses platelet activation by reducing the phosphorylation of phospholipase C (PLC) γ2, protein kinase C (PKC), and MAPKs induced by collagen phosphorylation. The above properties offer potential as a therapeutic approach for thromboembolic disorders [27]. Another study investigated the hepatoprotective effects of bioactive compounds obtained from 50 Gentian species on aconitine-induced hepatotoxicity in the human liver cancer cell line HepG2 by analyzing molecular docking to the active sites of the human cytochrome P450 3A4 (CYP3A4) enzyme. Amarogentin showed the strongest inductive effect on CYP3A4 mRNA levels, leading to enhanced drug metabolism, reduced oxidative stress, and improved mitochondrial function [28].

#### 4.1.2. Gentiopicroside and Inflammatory Properties

Gentiopicroside is a secoiridoid (Table 1), whose anti-inflammatory properties were observed in a study about the influence of gentiopicroside on dextran sulfate sodium (DSS)-induced colitis in a mouse model. Gentiopicroside treatment significantly attenuated damage to the intestinal mucosa and symptoms, including weight loss and diarrhea. This was associated with decreased mRNA expression of inflammatory markers, as well as tumor necrosis factor-α (TNF-α), interleukin-1β (IL-1β), IL-6, a reduction in the overexpression of cyclooxygenase-2 (COX-2), and inducible nitric oxide synthase (iNOS) proteins in the colon [29]. Furthermore, another study demonstrated the anti-inflammatory properties of gentiopicroside, in which *Gentiana macrophylla* root extract was used in rat models of rheumatoid arthritis [30]. The main active component in *Gentiana macrophylla* is primarily gentiopicroside [31]. Orally administered root extract markedly lowered the prostaglandin E2 (PGE2) levels in inflammatory tissues compared to the control (saline) and prednisone groups, which suggests that the inhibition of the COX2 enzyme most likely occurred. Moreover, survival rates, after 28 days of treatment, were higher in the extract and control groups, whereas most rats in the prednisone group did not survive [30].

The root extract of *Gentiana manshurica*, with confirmed presence of gentiopicroside, proved useful in treating alcohol-induced liver steatosis in mice. The observed effects included protection against an increase in malondialdehyde (MDA) levels, restoration of glutathione (GSH) levels, and increased activities of superoxide dismutase (SOD), catalase (CAT), and glutathione peroxidase (GPX). Furthermore, *Gentiana manshurica* attenuated the nuclear translocation of sterol regulatory element-binding protein-1 (nSREBP-1) and suppressed CYP2E1 induction, suggesting it prevents ethanol-induced liver damage by reducing fatty acid synthesis and oxidative stress [32]. In vivo models of acute and chronic alcoholic hepatosteatosis showed that gentiopicroside upregulates the (liver kinase B1) LKB1 and (5′AMP-activated protein kinase) AMPK pathways and downregulates SREBP1 and peroxisome proliferator-activated receptor α (PPARα), leading to attenuation of lipogenesis and increased lipid oxidation in ethanol-exposed cells. Gentiopicroside can modulate the LKB1/AMPK pathway by suppressing the activation of the P2X7 receptor-NLRP3 inflammasome, thereby inhibiting IL-1β production. Its anti-inflammatory properties and regulation of lipid metabolism highlight its hepatoprotective pharmacological potential for treating alcoholic hepatosteatosis [33]. Gentiopicroside provides significant hepatoprotection, which is associated with its anti-apoptotic properties. The inhibition of JNK/ERK MAPK signaling is also believed to contribute to the anti-apoptotic effects of gentiopicroside [34]. Gentiopicroside was also effective in the treatment of severe cholestasis and liver injury in mice. Pretreatment improved bile acid metabolism and reduced the intracellular bile acid pool back to basal levels. Gentiopicroside significantly upregulated hepatic mRNA levels of bile acid synthesis enzymes (Cyp8b1 and Cyp27a1), efflux transporters (Mrp4, Mdr1, and Ost-β), and ileal bile acid circulation mediators (Asbt and Fgf15). This was associated with a decrease in serum hepatic bile acid levels, as well as an increase in urinary and fecal bile acid excretion [35]. Gentiopicroside’s hepatoprotective properties are associated with its anti-apoptotic properties. The inhibition of JNK/ERK MAPK signaling is also believed to contribute to the anti-apoptotic effects of gentiopicroside [34]. Gentiopicroside can reduce both inflammation and fibrosis in the lungs of mice with pulmonary fibrosis (PF). It decreases levels of TNF-α and IL-1β in bronchoalveolar lavage fluid and decreases the hydroxyproline content in the lungs. Furthermore, gentiopicroside downregulates the expression of TGF-β1 and connective tissue growth factor (CTGF) in the lungs and also inhibits the epithelial-mesenchymal transition of A549 cells induced by TGF-β1 [36]. Additionally, gentiopicroside may be useful in osteoporosis treatment through its impact on receptor activator of nuclear factor-κB ligand (RANKL)-induced osteoclastogenesis. Gentiopicroside inhibited the activation of JNK and NF-κB signaling pathways, thereby preventing osteoclastogenesis [37]. Gentiopicroside is also being investigated for its potential to reduce neurotoxicity by modulating astrocyte-mediated inflammation. Gentiopicroside reduced the release of TNF-α, IL-1β, nitric oxide (NO), and PGE, as well as the expression of iNOS and COX-2 in astrocytes. It also suppressed NF-κB translocation and JNK/SAPK MAPK phosphorylation, while having minimal effect on p-p38 levels [38]. Also, the secoiridoid glycoside cruciatoside, isolated from the aerial parts of *Gentiana cruciata* L., has demonstrated both anti-inflammatory and analgesic activities. Its anti-inflammatory effect involves the inhibition of nitrite production in lipopolysaccharide (LPS)-induced RAW 264.7 macrophage cells, along with a decrease in IL-6 levels. The analgesic effect is attributed to the reduction in prostaglandin E2 production [39].

#### 4.1.3. Swertiamarin—Neuro- and Hepatoprotection

Swertiamarin (Table 1) is an active secoiridoid glycoside compound from *Gentiana* species, which is reported to reduce cerebral ischemic/reperfusion (I/R) injury through the suppression of oxidative stress, and could potentially find application in the treatment of ischaemic stroke. The action mechanism is based on the activation of the Nrf2 protective pathway, which inhibits reactive oxygen species (ROS) associated with NF-ĸB activation. Swertiamarin pretreatment enhanced nuclear factor erythroid 2-related factor 2 (Nrf2) nuclear translocation from Kelch-like ECH-associated protein 1 (Keap1)-Nrf2 complex. It increased the expressions of nicotinamide adenine dinucleotide phosphate hydrogen (NAD(P)H): quinone oxidoreductase-1 (NQO1) and heme oxygenase-1 (HO-1), both in vivo and in vitro [40,41]. A study based on a rat model of hepatic fibrosis induced by dimethylnitrosamine (DMN) demonstrated the potential of swertiamarin as a treatment for hepatic fibrosis by targeting the renin-angiotensin system (RAS). Swertiamarin was found to lower plasma levels of angiotensin II (Ang II) and inhibit the activation and proliferation of HSCs, leading to a decrease in fibrosis markers such as α-SMA and collagen deposition in the liver. Furthermore, swertiamarin attenuated the upregulation of angiotensin type 1 receptor (ATR1), and it also inhibited the phosphorylation of ERK and c-Jun, which are induced by Ang II and, in this case, caused by DMN exposure [42]. Another study that investigated the hepatoprotective properties of swertiamarin showed that it induces the expression of CYP2E1, CYP3A, efflux transporters, and PDZ domain containing 1 (PDZK1), partially through the Nrf2/HO-1 pathway, which results in more efficient hepatic detoxification. Furthermore, suppression of the inflammatory response and hepatic oxidative stress was illustrated by a decrease in the levels of iNOS, IL-1β, and MDA [43]. Swertiamarin was also shown to be effective against D-galactosamine-induced liver injury in rats. Treatment resulted in a significant increase in the levels of antioxidant enzymes, including CAT, SOD, and GSH, while decreasing the levels of thiobarbituric acid-reactive substances (TBARS) in the serum, liver, and kidneys. Histological analysis revealed less liver necrosis and bile duct proliferation in the treated groups [44].

#### 4.1.4. Luteoloside—Autophagy-Mediated Anticancer Effects

Luteoloside (Table 1) is a flavonoid from *Gentiana macrophylla* that shows anti-cancer effects in non-small cell lung cancer (NSCLC) by inducing autophagy rather than apoptosis and suppressing growth pathways. Autophagy was evidenced by the formation of autophagic vacuoles, degradation of p62, and increased expression of Bcl-2 interacting protein (Beclin-1), and microtubule-associated protein 1A/1B-light chain 3-phosphatidylethanolamine conjugate (LC3-II). The above effects were observed in A549 and H292 cells but were absent in BEAS-2B cells. Luteoloside affects the crucial autophagy-suppressive cascade, the PI3K/AKT/mTOR/p70S6K pathway by inhibiting phosphorylated-Akt (p-Akt), the phosphorylated-mammalian target of rapamycin (p-mTOR), and p-p70S6K. Furthermore, it was observed that luteoloside-induced cell autophagy was associated with the production of ROS. The results additionally showed that ROS is an effector that increases the activity of the PI3K/AKT/mTOR/p70S6K pathway. Moreover, luteoloside inhibits NSCLC cell proliferation and causes G0/G1 phase arrest by attenuating the expression of CyclinE, CyclinD1, and Cyclin-dependent kinase 4 (CDK4) [45]. In contrast, another study showed that luteoloside treatment significantly reduced intracellular ROS accumulation in hepatocellular carcinoma (HCC) cells. This reduction in ROS was associated with decreased expression of the multi-protein complex, NLRP3 inflammasome, leading to attenuated activation of caspase-1 and reduced secretion of IL-1β. These findings suggest that luteoloside’s anti-cancer effects in HCC cells may be mediated through its antioxidative properties and suppression of inflammatory pathways. It has been demonstrated that luteoloside inhibits the proliferation, invasion, and metastasis of HCC both in vitro and in vivo [46].

#### 4.1.5. Isovitexin—Anti-Atherosclerotic Potential

Isovitexin (Table 1) is a flavonoid that could be a promising candidate for atherosclerosis prevention and treatment [47]. A study using *Gentiana autumnalis* root extract containing isovitexin showed that it prevents the proliferation of aortic smooth muscle cells (SMCs) induced by platelet-derived growth factor (PDGF)-BB, a significant factor in atherosclerosis. Isovitexin suppresses the activation of ERK1/2, a critical pathway involved in cell proliferation. Therefore, prevents PDGF-BB-induced expression of iNOS and reduces NO levels. Docking analysis suggests that isovitexin binds to the MEK1 kinase, inhibiting ERK1/2 activation [48]. Another study that analyzed isovitexin as a candidate for atherosclerosis treatment revealed additional beneficial effects, such as a reduction in total cholesterol levels in the blood of supplemented rats, reduced expression of vascular cell adhesion molecule 1 (VCAM-1), iNOS, and vascular endothelial cadherin (VE-cadherin) in the aortic segments of diabetic rats. Moreover, isovitexin was found to attenuate PDGF-BB-induced ROS and calcium rise, which are vital for SMCs migration, contributing to the plaque volume [49].

#### 4.1.6. Isoorientin—Anti-Melanogenic Activity

Isoorientin (Table 1) is a major flavone in the extract from *Gentiana veitchiorum* that could be useful in the treatment of pigmentary skin disorders due to its inhibitory effects on melanogenesis in B16F10 cells. Isoorientin attenuates the expression of vital melanogenic enzymes, including tyrosinase (TYR), tyrosinase-related protein-1 (TRP1), and DOPA-chrome tautomerase (DCT). Moreover, isoorientin inhibits the expression of microphthalmia-associated transcription factor (MITF) by promoting the phosphorylation of cyclic adenosine monophosphate (cAMP) response element-binding protein (CREB) [21].

#### 4.1.7. Gentiolactone—Targeted Anti-Inflammation

Gentiolactone (Table 1) is a secoiridoid dilactone that has anti-inflammatory properties. In a study using RAW264.7 cells as a model, gentiolactone isolated from the root extract of *Gentiana triflora* suppressed lipopolysaccharide (LPS)-induced expression of TNF-α, whereas swertiamarin and gentiopicroside, which were also included in the extract. Resulting in the attenuation of LPS-induced iNOS and COX-2 expression at the mRNA level. Furthermore, gentiolactone inhibits NF-κB transcriptional activity without suppressing NF-κB nuclear transport and without stimulating IκB degradation [50].

#### 4.1.8. Bellidifolin—Neuroprotection in Ischemia

Bellidifolin (Table 1) is a xanthone contained in plants of the genus *Gentiana*. It is known for its neuroprotective properties against ischemia and sciatic nerve injury. A study investigating the impact of pretreatment with bellidifolin on the severity of nerve injury caused by hypoxia showed a significant increase in the survival rate of pheochromocytoma cells (PC12) in the study group. Bellidifolin reduced hypoxia-induced expression of phosphorylated p38 MAPK (p-p38 MAPK) and caspase-3, thereby reducing apoptosis in hypoxia-induced nerve injury. Therefore, the pharmacological properties of this substance could potentially have therapeutic applications in the treatment of stroke [51].

### 4.2. Chemical Composition and Pharmacological Significance

In addition to iridoids, xanthones, and flavonoids, *Gentiana* plants also contain other bioactive compounds. Phytochemical analysis of the extract from *Gentiana spicata* revealed a high phenolic content. It resulted in the isolation and identification of a new compound, carboxygentisic acid (also proposed to be named spicatic acid), which is a 1,4-dicarboxy 2,5-dihydroxybenzene. This compound demonstrates potential hepatoprotective activity [3].

Gentian herbs, particularly *Gentiana lutea* and other species like *Gentiana scabra*, have long been associated with liver protection in traditional medicine. The liver-protective properties of *Gentiana* are primarily attributed to its ability to enhance bile production, support detoxification processes, and reduce liver inflammation through several active compounds. The hepatoprotective activities are related to sweroside, swertiamarin, and gentiopicrin—constituents of *Gentiana* root [52].

Another study demonstrated that the methanolic extract of *Gentiana manshurica* roots significantly reduced the elevated levels of serum aspartate aminotransferase (AST) and alanine aminotransferase (ALT), as well as serum and hepatic triglycerides, in ethanol-treated C57BL/6 mice. Additionally, it showed the ability to prevent alcohol-induced acute liver steatosis by inhibiting CYP2E1-mediated free radical production and suppressing the synthesis of SREBP-1 [53,54].

It is worth adding that Gentian’s primary therapeutic effects are linked to its ability to stimulate the production of gastric juices, digestive enzymes, and bile. Some studies revealed that Gentiopicroside manifested prokinetic activity in rats with gastrointestinal motility disorders induced by stress. The underlying mechanism involved the regulation of somatostatin and gastrin levels in plasma, increasing the expression of plasmatic motilin receptor in the gastric antrum, duodenum, ileum, and jejunum, and decreasing the expression of vasoactive intestinal Peptide receptor 2 in the duodenum [55]. As mentioned before, the gentiopicroside treatment can have anti-inflammatory effects in experimental acute colitis by reducing the expression levels of TNF-α, IL-1β, IL-6, iNOS, and COX-2, suggesting its potential therapeutic application in the treatment of colitis [29]. Moreover, herbal products derived from *Gentiana* spp. roots are very effective for improving appetite [56]. Another study claims that secoiridoidal glycosides isolated from different *Gentiana* species have several important activities. Amarogentin and amaroswerin have the strongest gastroprotective effects among the other secoiridoidals [57].

Additionally, the *Gentiana* species and their constituents affect many different factors related to vascular disease development and its progression. Research has shown that gentian herbs possess various pharmacological properties, such as anti-inflammatory, antioxidant, and vasodilatory effects, which play a key role in improving vascular health. Studies suggest that gentiana-based therapeutics represent potentially useful drugs for the management of vascular diseases [58]. What is more, isoorientin, derived from flowers of *Gentiana veitchiorum*, was found to inhibit melanogenesis in B16F10 murine melanoma cells. This suggests its potential as a skin-lightening agent for the treatment of skin pigmentary disorders [21]. Moreover, another study claims that *Gentiana lutea* extract might be used to improve some skin disorders with an impaired epidermal barrier, very dry skin, and atopic eczema [59].

Extracts from *Gentiana lutea* were found to have the potential to protect human vascular endothelial cells from damage caused by cigarette smoke. Isogentisin, isolated from this extract, also promotes cell survival by activating cellular repair mechanisms [60]. In addition, gentiopicroside holds promise as an ideal drug candidate for pulmonary fibrosis due to its anti-inflammatory and anti-fibrotic effects. In this context, alveolar epithelial cells and TGF-β may serve as the primary target cells and molecules for gentiopicroside in the treatment of bleomycin-induced pulmonary fibrosis [36]. In addition, the current study revealed that gentiopicroside exhibited a strong protective effect against inflammation induced by IL-1β in rat articular chondrocytes. This suggests its potential as a therapeutic strategy for osteoarthritis, as it not only alleviates inflammatory responses but also promotes the increased expression of Collagen type II, a key component of cartilage. By enhancing Collagen type II production, gentiopicroside may help in maintaining cartilage integrity and potentially slowing the progression of osteoarthritis [61].

Finally, the studies have shown that the crude extract of *Gentiana lutea* has the potential to prevent reproductive toxicity and oxidative damage in rat testes caused by a high acute dose of ketoconazole, an antifungal drug known for its reproductive toxic effects. The active compounds in *Gentiana lutea*, such as those with antioxidant and anti-inflammatory properties, likely contribute to this protective effect by neutralizing the oxidative stress and inflammation induced by ketoconazole. *Gentiana lutea* could provide a promising approach to protect humans from testicular damage induced by ketoconazole, potentially leading to the development of a combined therapeutic strategy [62].

## 5. Therapeutic Properties of Gentian Herbs

Gentiana plants, commonly referred to as Gentians, have been utilized for centuries in traditional medicine systems, such as Tibetan and Chinese medicine, due to their pronounced anti-inflammatory properties. These plants are particularly noted for their protective effects on liver and lung function, including the prevention of fibrosis development [63]. Additionally, Gentians have been employed in the treatment of inflammatory conditions, including rheumatoid arthritis and ulcerative colitis [64].

The primary compounds responsible for the anti-inflammatory and hepatoprotective effects of *Gentiana* plants are iridoids and secoiridoids [65]. Among these, the iridoid gentiopicroside has demonstrated significant therapeutic potential, exhibiting anti-rheumatic activity and anti-inflammatory effects in rat articular chondrocytes [61,66]. Iridoids are a class of monoterpenes characterized by a cyclopentane ring fused with a six-membered oxygen-containing heterocycle. Their biological effects are primarily attributed to their ability to modulate cytokine release in cellular models and their involvement in pathways mediated by nitrite and IL-6 [67,68]. Examples of iridoids isolated from plants of the genus *Gentiana* include swertiamarin, sweroside, and amarogentin, notably from *Gentiana rigescens* and *Gentiana cephalantha*, as well as gentiridoid A, B, C, D, and E, identified in *Gentiana decumbens*, *Gentiana dahurica*, and *Gentiana macrophylla* [69,70]. Secoiridoids are synthesized through the iridoid biosynthetic pathway. This process involves the production of geraniol from isoprenoid units via the MEP (2-C-methyl-D-erythritol-4-phosphate) pathway, followed by its conversion into loganinate and secologanin, which serve as direct precursors of secoiridoids. These conversions are catalyzed, among others, by the enzyme secologanin synthase (SLS) [71,72]. Secoiridoids, such as gentiopicroside and depressin, have demonstrated the ability to inhibit the production of proinflammatory cytokines, including IL-6 and TNF-α [68]. In studies conducted on LPS-induced RAW264.7 macrophage cells, compounds like oliveroside B exhibited significant anti-inflammatory activity by suppressing cytokine production [73]. Additionally, gentiopicroside was shown to modulate the CD147/P38/NF-κB signaling pathway, a key regulator of inflammatory responses [66].

Plants of the *Gentiana* genus demonstrate significant antioxidant activity, as confirmed by various studies examining different species within the genus. For instance, extracts derived from both the root and aerial parts of *Gentiana lutea* have been identified as effective radical scavengers [74]. Similarly, an ethanol extract from *Gentiana septemfida* exhibited a high capacity to neutralize free radicals. The bioactive compounds responsible for the antioxidant properties of *Gentiana* species predominantly include phenolic compounds such as ferulic acid, ellagic acid, and 4-hydroxybenzoic acid; iridoids; flavonoids such as luteolin, apigenin, and kaempferol; xanthones such as gentisin and isogentisin, and other compounds like gentiascabraside A and gentiatibetin [69,75,76,77].

## 6. Digestive Health Benefits—Potential Role in Liver Protection and Detoxification

To start with, *Gentiana* herbs are traditionally used to treat various gastrointestinal problems, including stomachaches, heartburn, gastritis, diarrhea, and vomiting. As mentioned earlier, gentian root is used to treat functional dyspepsia, liver and gallbladder dysfunction, and also exhibits anti-ulcerogenic, antimicrobial, analgesic, radioprotective, choleretic, anti-inflammatory, and hypoglycemic properties. The Gentianaceae family is also used to treat eating disorders such as anorexia [78,79,80].

Several species from the Gentianaceae family have potential medical benefits, one of which is *Swertia chirayita*. *S. chirayita* can be used in addition to the treatment of liver diseases due to its hepatoprotective role associated with its antioxidant activity, as its metabolites are xanthones, seco-iridoids, terpenoids, alkaloids, and flavonoids [81,82]. The study revealed that *S. chirayita* is hepatoprotective, especially after the administration of hepatotoxic paracetamol. The Swiss albino mice were administered a 150 mg/kg dose of paracetamol and 100–200 mg/kg extract of *S. chirayita*. The result showed that the addition of *S. chirayita* extract helped to lower the levels of serum enzymes that were elevated by paracetamol, such as GPX, CAT, alkaline phosphatase (ALP), and lipid peroxides (LPO), leading to the conclusion that *S. chirayita* protected the liver cells from hepatotoxicity from administered doses of paracetamol [83]. Another important medical use of *Swertia chirayita* is its anti-hepatitis B activity. The researchers discovered that compounds isolated from *S. chirayita* block the secretion of hepatitis B surface antigen (HBsAg) and hepatitis B e antigen (HBeAg), and also inhibit hepatitis B virus (HBV) DNA replication. The anti-hepatitis B activity sounds very promising; however, the studies should be expanded in the future, as there is still no clear information about the components responsible for anti-HBV activity [84].

As mentioned earlier, several species from the Gentianaceae family have been used in traditional medicine, including those in Asian countries. *Gentiana* species have been traditionally used in Chinese medicine for the treatment of digestive, liver, or gallbladder diseases [2]. A Tibetan plant called Jie-Ji was used to reduce the symptoms of so-called Chi-Ba disease, manifested by abdominal swelling, and jaundice symptoms such as yellowness of eyes, skin, and a darker shade of urine [85]. *Gentiana manshurica* and its root, known as gentianae radix, were also used in traditional Chinese medicine as a treatment for individuals with liver diseases. One of them is acute alcohol-induced fatty liver, where elevated serum levels of AST, ALT, and serum and hepatic levels of triglycerides can be observed. The administration of *Gentiana manshurica* successfully decreased these parameters and also offered protection from hepatic steatosis and necrosis induced by alcohol [32,53]. The studies showed that *Gentiana manshurica* is as hepatoprotective as previously mentioned *S. chirayita* against the hepatotoxic acetaminophen (also called paracetamol) [86]. Mice were injected with 300 mg/kg body weight of acetaminophen and orally administered *Gentiana manshurica* (200, 150, or 50 mg/kg body weight). As a result, the serum levels of AST and ALT were increased in comparison to mice that were not given the dose of acetaminophen. What is more, the mice who were administered *Gentiana manshurica* presented decreased ALT and AST serum levels—the ALT serum levels were reduced to 43%, 26%, 13%, or 13% in mice who were administered *Gentiana manshurica* (200, 100, and 50 mg/kg) and AST serum levels also decreased to 58%, 14%, 10%, or 8% in mice who were given *Gentiana manshurica* (200, 100, and 50 mg/kg). Moreover, the injection of acetaminophen also caused a significant decrease in hepatic GSH, GPX, and SOD activity. The administration of *Gentiana manshurica* restored the levels of the given parameters, protecting the liver from acetaminophen-induced oxidative stress [87].

As was recently mentioned, *Gentian* herbs have hepatoprotective properties due to the presence of monoterpenes, gentiopicroside, sweroside, and swertiamarin. The study showed that sweroside reduces the level of ALT in patients who suffer from hepatitis. The methanol extracts of *Gentiana cruciata* aerial parts and roots, containing high levels of sweroside and gentiopicroside, are useful in the treatment of liver injuries triggered by carbon tetrachloride. These extracts normalize the elevated serum levels of AST, ALT, and ALP [88,89,90]. The facilitation of drug metabolism causes hepatoprotective effects, amelioration of mitochondrial dysfunction, and a reduction in oxidative stress [28]. *Gentiana macrophylla* could also decrease the activities of ALT and AST in the serum of CCl4-induced acute liver injury mice, as well as MDA in the liver, and it also enhances the activity of SOD. Following the administration of this extract, the damaged hepatic tissue structure was restored, and the amount of collagen deposition decreased. In addition, the liver index and body weight were improved [91,92]. The same effect was observed after the administration of methanol extract from *Gentiana cruciata* aerial parts and roots, as it also attenuates carbon tetrachloride-induced liver injury in Wistar rats, causing decreased levels of transaminases, ALP, and total bilirubin in serum. Additionally, increased levels of SOD, CAT, and GSH were observed as well [52,88].

The gentiopicroside from *Gentiana macrophylla* attenuates the chloroform and LPS/bacillus Calmette–Guérin-induced liver injuries in mice. The researchers examined the influence of *Gentiana macrophylla* ethanol extract on the hepatic tissue damaged by parvovirus B19- non-structural protein (NS1)-exacerbated liver injury in NZB/W F1 mice. The results revealed that the *Gentiana macrophylla* root extract significantly reduced the expression of urokinase plasminogen activator and hepatic matrix metalloproteinase-9 in response to B19-NS1-exacerbated liver inflammation [88,91,93].

*Gentiana lutea* is another species from the Gentianaceae family that exhibits gastroprotective activity. The study showed that the extract of gentian root offered protection against acute gastric ulcers in mice. Moreover, the secoiridoid glycosides isolated from the gentian roots, such as amarogentin, gentiopicroside, amaroswerin, and swertiamarin, inhibited 50% of stress-induced ulcers, and the suppressive effect on ethanol-induced gastritis reached even 45.4% [57]. This extract can also be used as a beverage ingredient with a proven gastroprotective effect. The beverage can be used, firstly, to promote stomach acid secretion; secondly, as a remedy for gastritis, and thirdly, in the treatment of ailments for patients suffering from a lack of appetite and flatulence. *Gentiana lutea* also affects the contractions of smooth muscle that form the ileum. The tests revealed that isogentisin, loganic acid, sweroside, and gentiopicroside from the gentian root relaxed the muscles during spontaneous contraction. This study also showed that gentiopicroside can reduce contractions induced by histamine, acetylcholine, KCl, and BaCl_2_ [94]

Some studies revealed the gastroprotective effects of the methanol extract from gentian root in different gastric lesions. In pylorus-ligated rats, this extract suppresses the secretion of gastric juice and total acid output. This extract is also active against acute gastric ulcers induced by aspirin and gastric mucosal injury induced by ethanol [95]. A great example of a gastroprotective herb is *Gentiana macrophylla*. *Gentiana macrophylla* contains iridoids and flavonoids, which show gastrointestinal tract protective activity as they induce mucus, acid, and enzyme creation in the stomach. A study conducted on mice revealed that gentiopicroside, found in gentian herbs, protects the stomach mucosa from damage caused by ethanol. In the examination of rats with gastrointestinal motility disorders, gentiopicroside accelerates intestinal motility and gastric emptying, increases gastrin levels, decreases somatostatin levels in plasma, inhibits the expression of vasoactive intestinal peptide 2 receptor in the duodenum, and increases the expression of motilin receptor in the pylorus of the stomach, duodenum, jejunum, and ileum [47,55,91,96].

Another herb used for the treatment of chronic stomach diseases is *Gentiana triflora,* as it reduces gastric acidity, inflammation, and cholecystitis. It is also used to improve appetite and cure digestive issues as the *Gentiana triflora* leaves contain isosreintin and 4-o-glucoside. A study has shown changes in stomach secretion after the administration of gentian herb extracts, including *Gentiana triflora*. Even though the best results were achieved after the administration of *Gentiana algida* extract, *Gentiana triflora* also showed a significant increase in gastric juice volume, total and free hydrochloric acid concentration, and pepsin concentration. Other herbs, such as Gentiana decumbens and Gentiana macrophylla, showed weak activity in changing these stomach parameters [47,97].

## 7. Other Applications for Gentian Extracts in Medicine

### 7.1. Anti-Bacterial and Anti-Inflammatory Effects

It is worth noting that Gentiana lutea has an antibacterial effect, primarily due to its content of gentiopicrin, mangiferin, and isogentisin. The study that has proven these properties was conducted by testing 16 different pathogens, including *Escherichia coli*, *Salmonella typhimurium*, *Salmonella enteritidis*, *Pseudomonas aeruginosa*, *Staphylococcus aureus*, *Streptococcus faecalis*, *Listeria monocytogenes*, and *Candida albicans*. The *Gentiana lutea* extracts inhibited the growth of 15 pathogens, and only *Listeria monocytogenes* was resistant. Among the individual extracts of components, the compound with the widest spectrum of activity was gentiopicrin, and it was the most active and effective against *Escherichia coli* [2,98]. Additionally, the anti-inflammatory properties were demonstrated in an experiment on rats. The rats were administered the *Gentiana macrophylla* water extract orally at a daily dose of 100 mg/kg body weight. The prostaglandin E2 levels in the inflammatory tissues, sole thickness, and ankle circumferences of the feet were significantly decreased. The water extract of *Gentiana macrophylla* is also likely to increase the level of IL-1 [30,91,96]. Some studies have revealed that the anti-inflammatory effects are a result of inhibiting IL-6, thanks to gentiopicroside, sweroside, and swertiamin [77,88]. The 3-day treatment with gentiopicroside significantly attenuates 70% ethanol-induced gastric mucosal injury in mice, likely due to the reduction in cytokines and oxidative stress [88,99]. Table 2 below collects the most well-known properties of Gentiana species.

### 7.2. Appetite Stimulation

As mentioned before, *Gentiana lutea*, also known as the yellow gentian, was used as a cure for temporary loss of appetite [103]. Furthermore, the study demonstrates that bitter-tasting compounds from *Gentiana lutea* modulate eating behavior through bitter-taste receptors in the gastrointestinal tract. Some studies revealed that the activation of bitter receptors in the gastrointestinal tract may result in the secretion of hormones modulating appetite and eating behavior [105]. The study revealed that administration of bitter substances immediately increases the release of ghrelin, which causes the increased food intake during the first 30 min of the test, followed by the inhibition of stomach emptying [100]. This effect was observed not only because of the inhibitory effect of ghrelin but especially as a result of the blockage of bitter taste receptors. *Gentiana lutea* contains bitter gentiopicroside, which has a significant effect on improving appetite loss. The hydroalcoholic solution prepared from the extract of this herb contains a high concentration of gentiopicroside. In studies, gentiopicroside has been shown to improve appetite, potentially leading to weight gain due to increased food intake. However, the study proved that the concentration of gentiopicroside was higher in a solution prepared from *Gentiana olivieri* than in a solution prepared from *Gentiana lutea* [108].

## 8. Discussion

Gentianaceae species, particularly those from the genus Gentiana, for a long time have played an important role in traditional medicine in Asia, Europe, and the Middle East. Their usage has a wide spectrum of conditions, including digestive disorders, liver ailments, metabolic diseases, inflammatory conditions, and wound healing [90,109]. Over recent decades, modern pharmacological research has begun to validate many of these traditional applications by uncovering the bioactive roles of key phytochemicals—primarily seco-iridoids, flavonoids, and xanthones—and their mechanisms of action. In this review, we synthesized the therapeutic relevance of Gentianaceae extracts based on both traditional uses and preclinical/clinical evidence, with emphasis on signaling pathways, targeted organs, and emerging applications in metabolic, cardiovascular, and autoimmune diseases. What is important is that clinical and experimental studies strongly correlate with traditional medicine observations and place the growing pharmacological foundation behind these age-old remedies.

The extract of *Gentiana lutea* exhibits ultraviolet (UV) protective and antioxidant properties, which may help protect the skin from DNA damage and oxidative stress. Additionally, it has the potential to prevent skin diseases, including cancer, due to its anti-genotoxic properties [106,107]. Gentiana is used in traditional Chinese medicine for the treatment of rheumatism, hepatitis, and pain. These plants also exhibit antifungal properties due to the presence of bisphosphocholines, which may help to combat fungi such as Candida and Aspergillus [107]. Extracts of *Gentiana lutea* exhibit inhibitory activity against aldose reductase (ALR2), which may prevent or delay diabetic complications, such as sorbitol accumulation in cells. Additionally, compounds like amarogentin may serve as a potential therapy to support diabetes treatment [102]. Other herbs that can be used in the treatment of diabetes include Gentiana dinarica and Gentiana utriculosa, as they inhibit the intestinal α-glucosidase enzyme, which slows down the digestion and absorption process of complex carbohydrates. In turn, they alleviate the increase in postprandial glycemia [110]. Extracts from the roots of *Gentiana lutea* prevent the proliferation of aortic SMCs by blocking cellular responses triggered by PDGF-BB. PDGF-BB stimulates the proliferation of these cells, which can contribute to the development of atherosclerosis. *Gentiana lutea* extracts inhibit this process by blocking the activation of the ERK1/2 signaling pathway and reducing the levels of NO in the cells. Additionally, these extracts suppress the expression of genes associated with proliferation, such as iNOS, cyclin D1, and proliferating cell nuclear antigen (PCNA). As a result, *Gentiana lutea* may have the potential to prevent the development of atherosclerosis and other diseases related to the uncontrolled proliferation of SMCs [48]. The extract of *Gentiana lutea* demonstrates anti-obesity properties, making it potentially useful in the prevention and treatment of obesity. In vitro studies on 3T3-L1 fat cells showed that the extract inhibits adipocyte differentiation by reducing the expression of adipogenesis-related genes and lipid accumulation. In studies on a mouse model of obesity induced by a high-fat diet (HFD), the extract prevented weight gain, reduced adipocyte size, and minimized fat deposition in the liver. It also lowered leptin and insulin levels, indicating its beneficial effect on regulating lipid and glucose metabolism. Thus, Gentian extract may aid in preventing and treating obesity, making it a valuable component in the therapy of metabolic disorders [104].

Gentians are commonly used in traditional medicine for wound healing, especially in regions of the Middle East, the Caucasus, and the Mediterranean, including Iran, Turkey, Georgia, Bulgaria, Greece, and the Cantabrian Mountains. In Bulgaria, *Gentiana cruciata* is used in the form of poultices to treat eczema, ulcers, boils, purulent wounds, burns, and difficult-to-heal wounds. At the same time, *Gentiana lutea*, *Gentiana punctata*, and *Gentiana asclepiadea* are listed as alternative species. In traditional Iranian medicine, a poultice made from *Gentian* root mixed with vinegar is applied to infected wounds, as well as for treating other conditions such as urinary retention, menstrual disorders, liver and spleen problems, and detoxifying animal toxins (e.g., from scorpion and viper bites). The Gentian species mentioned in this context include *Gentiana olivieri*, *Gentiana gelida*, *Gentiana septemfida*, and *Gentianella caucasica*. Additionally, *Gentiana septemfida* is commonly used in Georgia to treat hemorrhoids, and in Turkey, several other *Gentian* species are also used for this purpose [6].

The roots of *Gentiana macrophylla,* known as Qinjiao, have been used for centuries to treat systemic lupus erythematosus (SLE). This study aimed to investigate whether *Gentiana macrophylla* exhibits anti-apoptotic effects in cholesterol-induced cardiac abnormalities associated with SLE. The study was conducted on NZB/W F1 mice fed a high-cholesterol diet, evaluating the morphology of the left ventricle (LV) tissues and apoptotic biomarkers. The results showed that *Gentiana macrophylla* significantly reduced cholesterol-induced apoptosis in the LV by inhibiting apoptotic pathways and increasing insulin-like growth factor 1 (IGF-1) survival signaling and anti-apoptotic proteins. *Gentiana macrophylla* shows potential in alleviating cholesterol-induced cardiac damage in SLE, offering an alternative treatment for SLE patients with cardiac abnormalities [111]. The classic role of Gentiana lutea root as a “stomach bitter” is backed by a human cross-over study showing that micro-encapsulated seco-iridoids lowered 24-h energy intake and modulated gut hormone release after a test meal [101,105]. The centuries-old prescription of Radix Gentianae for “liver heat” is underpinned by a CCl_4_-injury rat model in which an aqueous extract normalized transaminases, suppressed TNF-α/IL-6 and corrected metabolic fingerprints, confirming hepatoprotection [43]. Traditional use of Gentiana scabra in Korean medicine for diabetes is also linked to data showing its root extract boosts GLP-1 secretion and lowers blood glucose in db/db mice [112]. Moreover, reports that gentian tonics “strengthen the gut” are compatible with evidence that gentiopicroside tightens epithelial junctions and reshapes the microbiome in diet-induced NAFLD mice, thereby strengthening the intestinal barrier [113]. Anti-inflammatory poultices prepared from Swertia chirayita were echoed by xanthones that block COX-2 and NF-κB/MAPK/Akt signaling in LPS-stimulated macrophages [114]. Finally, the bone-strengthening reputation of Gentiana macrophylla is correlated with an ovariectomized mouse study, wherein its root extract prevented osteoporotic bone loss [93]. Together, these examples show how the family’s iridoids, flavonoids, and xanthones converge on digestion, hepatoprotection, glucose control, barrier integrity, and inflammation—yet, wild populations of flagship species such as G. lutea are already threatened by overharvesting, underscoring the need for conservation-minded sourcing [6]. Table 3 below summarizes the potential activity of Gentiana species.

Despite promising pharmacological profiles, the therapeutic use of Gentiana species remains constrained by limited clinical validation, the potential for toxicity at high doses, and the risk of drug–herb interactions—particularly with drugs metabolized by hepatic or glucoregulatory pathways (e.g., gentiopicroside and glucose control) [64]. Additionally, overharvesting of wild populations, especially flagship species like G. lutea and regionally used taxa such as G. dinarica and G. utriculosa, raises urgent conservation concerns and emphasizes the need for sustainable cultivation and ethical sourcing practices [6,11,69]. Future research should prioritize the standardization and quality control of extracts, rigorous evaluation of safety and herb–drug interaction potential in clinical trials, and innovative approaches to developing synergistic phytopharmaceutical formulations that maximize therapeutic benefit while preserving biodiversity.

## 9. Conclusions

The purpose of this review was to comprehensively evaluate the therapeutic potential of Gentiana species by examining their traditional uses, phytochemical constituents, and the current state of pharmacological research. By focusing on the most bioactive compound classes—iridoids, xanthones, and flavonoids—we aimed to bridge ethnomedicinal knowledge with mechanistic insights, highlighting how these compounds contribute to anti-inflammatory, hepatoprotective, metabolic, dermatological, and wound-healing activities [115,116]. This study is important because it combines the knowledge across botanical, pharmacological, and ethnomedical literature into a coherent framework, thus facilitating the rational development of Gentiana-based therapies. The species reviewed exhibit a wide range of clinically relevant bioactivities, including ultraviolet protection, anti-rheumatic, digestive, hepatoprotective, anti-diabetic, and anti-obesity effects, many of which are now supported by experimental and mechanistic studies.

Despite this promising potential, significant knowledge gaps remain—particularly in the areas of mechanisms of action, pharmacokinetics, metabolism, toxicology, and drug–herb interactions. These aspects must be rigorously addressed through well-designed in vitro, in vivo, and, finally, clinical studies to ensure both efficacy and safety. In conclusion, Gentiana species exhibit strong pharmacological potential with high scientific and therapeutic value. Their historical applications in traditional medicine, with current emerging experimental data, support their importance for further investigation as sources of novel plant-derived pharmaceuticals.

## Figures and Tables

**Table 1:** Representative 2D structures of bioactive molecules isolated from medicinal Gentianaceae species.

Compound	Structure
*Amarogentin* [11]	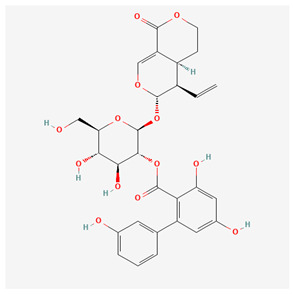
*Gentiopicroside* [12]	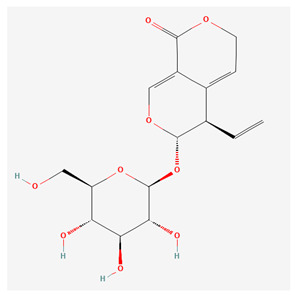
*Swertiamarin* [13]	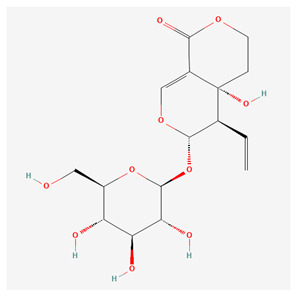
*Luteoloside* [14]	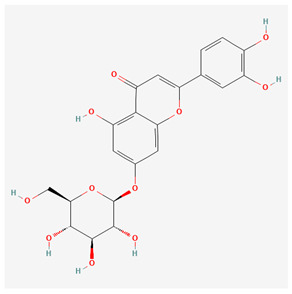
*Isovitexin* [15]	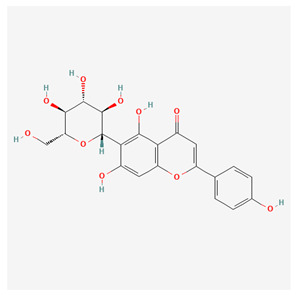
*Isoorientin* [16]	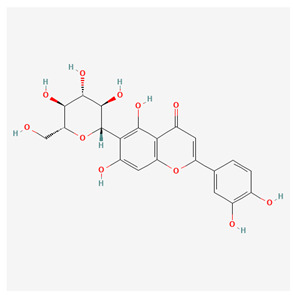
*Gentiolactone* [17]	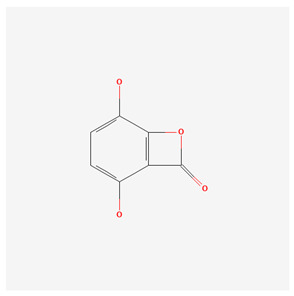
*Bellidifolin* [18]	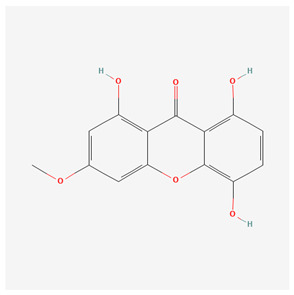

**Table 2:** Properties of *Gentiana* species.

*Gentiana* Species	Properties	References
*Gentiana macrophylla*	hepatoprotective activity	[88,91,92,93]
	anti-inflammatory function	[28,29,42,64,73,91]
	blood sugar rises	[93]
	gastrointestinal tract protection	[44,52,99]
	allergic shock prevention	[96,97]
	anti-histamine effect	[68]
	appetite stimulation	[66,67,100]
*Gentiana lutea*	spasmolytic effect on the gastrointestinal system	[94]
	appetite stimulation	[56,101]
	mild dyspepsia	[30,101]
	duodenal ulcer protection	[102]
	antipyretic effect	[57,103]
	wound healing	[2,71]
	hypoglycemic effect	[77]
	anti-anemic effect	[95,104]
	hepatoprotective effect	[55,105]
	skin disorders	[21]
	immune stimulant	[56]
	astringent activity	[94]
	antibacterial effect	[95,98,106,107]
*Gentiana scabrae*	stomach muscle strengthening	[66]
	liver protection	[66]
	cholagogic effect	[54]
	anti-inflammatory effect	[2]
	anti-bacterial effect	[91]
*Gentiana dinarica*	blood sugar level regulation	[93]
*Gentiana utriculosa*	blood sugar level regulation	[93]
*Gentiana cruciata*	appetite stimulation	[75]
	duodenal ulcer protection	[75]
	wound healing	[64]
	hepatoprotective effect	[13,37]
*Gentiana rigescens*	strengthening the stomach muscle	[66]
	liver protection	[66]
	cholagogue effect	[68]
	anti-inflammatory effect	[68]
	anti-bacterial effect	[68]
*Gentiana triflora*	chronic stomach disease treatment	[97]
	appetite stimulation	[44]
	jaundice treatment	[48]
	hepatitis treatment	[48]
*Gentiana punctata*	wound healing	[64]
*Gentiana* *asclepiadea*	wound healing	[64]
	appetite stimulation	[67]
	anti-anemic effect	[64]
	gall and liver disease treatment	[65]
*Gentiana* *septemfida*	hemorrhoids protection	[76]
	anti-inflammatory function	[60]
	wound healing	[64]
*Gentiana olivieri*	appetite stimulation	[53,70,108]
	wound healing	[64]

**Table 3:** Compounds present in *Gentiana* species and their activity.

Plant Parts	Compound	Functional Activity	Reference
root	amarogentin	anti-inflammatory, anti-bacterial, hepatoprotective, gastroprotective	[9,10,11,12,13,54]
root, aerial part	bellidifolin	neuroprotective properties against ischemia and sciatic nerve injury	[48]
root	bisphosphocholines	antifungal properties	[107]
root	carboxygentisic acid	hepatoprotective activity	[3]
root, aerial part	gentian violet	anti-bacterial, sepsis, meningitis	[111]
root	gentiolactone	anti-inflammatory	[49]
root	gentiopicroside	anti-inflammatory, anti-rheumatic, hepatoprotective, and preventing osteoclastogenesis	[14,15,16,17,18,19,20,21,22]
root, aerial part	isogentisin	cellular repair mechanisms, anti-bacterial	[2,78]
flowers	isoorientin	pigmentary skin disorders	[47]
leaves	isosreintin	chronic stomach diseases, appetite stimulation	[45]
root	isovitexin	atherosclerosis prevention, reduction in total cholesterol levels	[44,45,46]
root, aerial part	luteoloside	anti-cancer effect, antioxidative	[43,44]
root, aerial part	sweroside	hepatoprotective, antioxidative	[13]
root, aerial part	swertiamarin	hepatoprotective, reduction in cerebral ischemia, gastroprotective	[37,38,39,40,41,54]
